# Updating analysis of key performance indicators of 4G LTE network with the prediction of missing values of critical network parameters based on experimental data from a dense urban environment

**DOI:** 10.1016/j.dib.2022.108240

**Published:** 2022-05-05

**Authors:** Agbotiname Lucky Imoize, Samuel Oluwatobi Tofade, Glory Uzuazobona Ughegbe, Francis Ifeanyi Anyasi, Joseph Isabona

**Affiliations:** a Department of Electrical and Electronics Engineering, Faculty of Engineering, University of Lagos, Akoka, Lagos 100213, Nigeria; b Department of Electrical Engineering and Information Technology, Institute of Digital Communication, Ruhr University, Bochum 44801, Germany; c SeaArnaud Engineering Limited, Ikeja GRA, Lagos 100243, Nigeria; d Department of Electrical and Electronics Engineering, Faculty of Engineering and Technology, Ambrose Alli University, Ekpoma 310101, Nigeria; e Department of Physics, Federal University Lokoja, Lokoja, Nigeria

**Keywords:** Experimental dataset, 4G LTE network, Piecewise cubic hermite interpolating polynomial (PCHIP), Key performance indicators (KPI), Statistical error analysis, Probability density function, Missing data estimation errors, LTE, Long Term Evolution, PCHIP, Piecewise Cubic Hermite Interpolating Polynomial, GPS, Global Positioning System, QAM, Quadrature Amplitude Modulation, MIMO, Multiple Input Multiple Output, KPI, Key Performance Indicators, RSRP, Reference Signal Received Power, RSRQ, Reference Signal Received Quality, RSSI, Received Signal Strength Indicator, PCC, Primary Component Carrier, SINR, Signal-to-Interference-and-Noise Ratio, PCI, Physical Cell Identity, PUSCH, Physical Uplink Shared Channel, PDCP, Packet Data Convergence Protocol, E-UTRA, Evolved Universal Terrestrial Radio Access, EARFCN, E-UTRA Absolute Radio Frequency Channel Number, SCC, SecondaryComponent Carrier, PUCCH, Physical Uplink Control Channel, RLC, Radio Link Control, QoS, Quality of Service, UE, User Equipment, MAE, Mean Absolute Error, RMSE, Root Mean Squared Error, RAE, Relative Absolute Error

## Abstract

In practice, field measurements often show missing data due to several dynamic factors. However, the complete data about a given environment is key to characterizing the radio features of the terrain for a high quality of service. In order to address this problem, field data were collected from a dense urban environment, and the missing parameters were predicted using the Piecewise Cubic Hermite Interpolating Polynomial (PCHIP) algorithm. The field measurement was taken around Victoria Island and Ikoyi in Lagos, Nigeria. The test equipment comprises a Global Positioning System (GPS) and a Fourth Generation (4G) Long Term Evolution (LTE) modem equipped with a 2×2 MIMO antenna, employing 64 Quadrature Amplitude Modulation (QAM). The Modem was installed on a personal computer and assembled inside a test vehicle driven at a near-constant speed of 30 km/h to minimize possible Doppler effects. Specifically, the test equipment records 67 LTE parameters at 1 s intervals, including the time and coordinates of the mobile station. Thirty-two parameters were logged at 42,498 instances corresponding to 11 h, 48 min and 18 s of data logging on the mobile terminal. Sixteen important 4G LTE parameters were extracted and analyzed. The statistical errors were calculated when the missing values were exempted from the analyses and when the missing values were incorporated using the PCHIP algorithm. In particular, this update paper estimated the missing values of critical network parameters using the PCHIP algorithm, which was not covered in the original article. Also, the error statistics between the data (histograms) and the corresponding probability density function curves for the measured data with missing values and the data filled with the missing values using the PCHIP algorithm are derived. Additionally, the accuracy of the PCHIP algorithm was analysed using standard statistical error analysis. More network parameters have been tested in the update article than in the original article, presenting only basic statistics and fewer network parameters. Overall, results indicate that only the parameters which measure the throughput values follow the half-normal distribution while others follow the normal distribution.

**Specifications Table**

**Table:** 

Subject	Engineering and Technology
Specific subject area	Wireless Communications Engineering
Type of data	Table Chart Graph Figure
How the data were acquired	The data analyzed in this article were acquired through a drive test. A 4G LTE modem was installed on a computer and a Global Position System (GPS) were assembled in a vehicle and driven at 30 km/h. The measured data were logged at the one-second interval. The routes covered lie between Victoria Island and Ikoyi, Lagos State, Nigeria.
Data format	Raw Analyzed Filtered
Description of data collection	The 4G LTE Modem used for field data collection could record sixty-seven (67) parameters of the 4G LTE network. However, thirty-two (32) parameters were logged during the field data collection. Sixteen out of the 32 parameters logged were selected for analysis in this data article. The chosen parameters include; Reference Signal Received Power (RSRP), Reference Signal Received Quality (RSRQ), Received Signal Strength Indicator (RSSI), Primary Component Carrier Signal-to-Interference-and-Noise Ratio (PCC SINR), Physical Cell Identity (PCI), Downlink E-UTRA Absolute Radio Frequency Channel Number (DL EARFCN), 1st Secondary Component Carrier RSRP (SCC1 RSSP), SCC1 RSRQ, SCC1 RSSI, SCC1 PCI, SCC1 SINR, SCC1 DL EARFCN, PCC Physical Uplink Shared Channel (PCC PUSCH) Power, PCC Physical Uplink Control Channel (PCC PUCCH) Power, Packet Data Convergence Protocol (PDCP) Throughput for Downlink and LTE Radio Link Control (RLC) Throughput for Downlink. The acquired data were logged at 1 s intervals using a 4G LTE test Modem mounted on a computer housed in a test vehicle driven at 30 km/h. Test measurements were taken from evolved base stations (eNodeBs) in the investigated environments.
Data source location	Data were acquired from seven eNodeBs. Four are located in Victoria Island, Lagos, Nigeria (Lat 6.46648618, Long 3.38523924); (Lat 6.46581470, Long 3.39187876), (Lat 6.45051415, Long 3.38934411) and (Lat 6.44552333, Long 6.44552333) while the other three are located in Ikoyi, Lagos, Nigeria (Lat 6.43381161, Long 3.45678386), (Lat 6.42570328, Long 3.48156642) and (Lat 6.42803909, Long 3.49555505).
Data accessibility	The experimental dataset can be accessed using the following link: https://data.mendeley.com/datasets/78dhxwj56j/1 https://doi.org/10.17632/78dhxwj56j.1
Related data article	[1] A. L. Imoize, K. Orolu, and A. A.-A. Atayero, “Analysis of key performance indicators of a 4G LTE network based on experimental data obtained from a densely populated smart city,” *Data Br.*, vol. 29, no. 105304, pp. 1–17, 2020, https://doi.org/10.1016/j.dib.2020.105304.

**Value of the Data**

• The original data considered only six key performance indicators (KPIs) obtained from three sites, whereas sixteen KPIs have been tested in the updated data collected from seven eNodeBs. The updated data is robust and will aid in efficient network design and planning to ensure high quality of service for real-time wireless applications.
• The new data analysis provides a method of estimating missing values for different 4G LTE network parameters such as the RSRP, RSRQ, RSSI and others for the benefit of mobile subscribers and all parties in the wireless ecosystem.

## 1 Data Description

Radio propagation measurements of key performance indicators (KPI) in a typical wireless communication network are critical to assessing the quality of service (QoS) of a functional wireless network [1], [2], [3]. It is pretty challenging to obtain all measurements parameters with complete details in practice [4]. Some parameters are often not logged or missing from actual measurements due to a significant distance between the transmitter and receiver and other dynamic environmental factors. In order to estimate the missing values of these parameters, we employ the Piecewise Cubic Hermite Interpolating Polynomial (PCHIP) algorithm [5], [6], [7], [8].

Generally, LTE systems have eNodeBs that communicate with the user equipment (UE) [9], [10], [11]. Transferring data from eNodeBs to UEs is known as downlink transmission while moving data from UEs to the eNodeBs is known as uplink transmission [12]. There has been an increased deployment of real-time applications such as virtual meetings applications due to the impact of Covid-19 [13,14]. However, these applications require good QoS. In order to provide good quality of service for mobile subscribers, it is essential to determine the key parameters influencing the QoS [15], [16], [17]. To this end, this article analyses KPI parameters such as RSRQ, RSRP, RSSI, and thirteen others, as seen in the tested dataset [18]. Specifically, the dataset captured includes the Reference Signal Received Power (RSRP), Reference Signal Received Quality (RSRQ), Received Signal Strength Indicator (RSSI), Primary Component Carrier Signal-to-Interference-and-Noise Ratio (PCC SINR), Physical Cell Identity (PCI), Downlink E-UTRA Absolute Radio Frequency Channel Number (DL EARFCN), 1st Secondary Component Carrier RSRP (SCC1 RSSP), SCC1 RSRQ, SCC1 RSSI, SCC1 PCI, SCC1 SINR, SCC1 DL EARFCN, PCC Physical Uplink Shared Channel (PCC PUSCH) Power, PCC Physical Uplink Control Channel (PCC PUCCH) Power, Packet Data Convergence Protocol (PDCP) Throughput for Downlink and LTE Radio Link Control (RLC) Throughput for Downlink, and more.

This update paper focuses on estimating the missing values for each network parameter and evaluating the PCHIP algorithm used to predict the missing values via statistical error analysis. It is worth mentioning that only three site locations were tested in the original article, whereas seven eNodeBs have been investigated in the current paper. The existing article considered only six key performance indicators (KPIs), whereas several KPIs up to sixteen have been tested in the update article. Additionally, the updated report extracted and analysed sixteen important 4G LTE parameters. The methods used to produce the data in the update article slightly differ from the methods used to create the data in the related data article. Here, the data are logged at 1 s intervals, time-stamped, and thirty-two parameters were recorded every second, including the logging time and coordinates. Measured data were logged for a total number of forty-two thousand, four hundred and ninety-eight instances. This extensive measurement campaign produced better results than the limited logging methods applied in the original article. Also, the extensive logging and coverage indicate that the update data greatly complements the existing dataset. However, the new data do not invalidate the original dataset but show remarkable additional value.

Regarding the measurement equipment, a newer version of the 4G LTE Modem has been used in the updated measurement due to its fast processing capabilities. The new Modem has a higher upload speed and faster download processing time. Also, the new Modem is built with the Balong 5000 chipset, supporting carrier aggregation and enabling a 5G measurement campaign. The theoretical peak download speed of the Huawei Modem used in the updated article is doubled, reaching up to 3.6 Gbps compared to the one used in the initial measurements with LTE download speed up to 100 Mbit/s and LTE upload speed up to 50 Mbit/s. Other measurement tools used in the original experiment were maintained. The acquired data in the update article were analysed using MATLAB 2020a, whereas MATLAB 2018a was used in the initial analysis. The new features in the new MATLAB version also help simplify and fasten data processing.

The original article did not consider the missing values of key network parameters, which have been included in the update article. Specifically, we estimated the missing data using the Piecewise Cubic Hermite Interpolating Polynomial (PCHIP) algorithm, which was not covered in the original article. Also, we derived the error statistics between the data (histograms) and the corresponding probability density function curves for the measured data with missing values and the data where the missing values are filled using the PCHIP algorithm. Again, this aspect was not considered in the original article. Additionally, in the updated paper, the accuracy of the PCHIP algorithm was analysed using standard statistical error analysis, and more network parameters have been incorporated in the current investigation. These parameters could be further analysed by investigating the outliers of the processed, filled missing values and either exempting those values from the inquiry or further interpolating those outlier values. Again, this analysis extends the original article, presenting only basic statistics and fewer network parameters.

The KPI data would help in evaluating the performance of the network. The data would be very valuable to the network operators and the regulatory agencies for informed decision making. The data would help develop and test efficient algorithms to study critical performance indicators for emerging wireless communication systems. Also, the wireless community and all parties in the communication ecosystem will find the projected data useful for learning-based algorithmic development, network planning, design, implementation, optimization and management.

### 1.1 Drive Test and Data Exploration

**Fig. 1:**
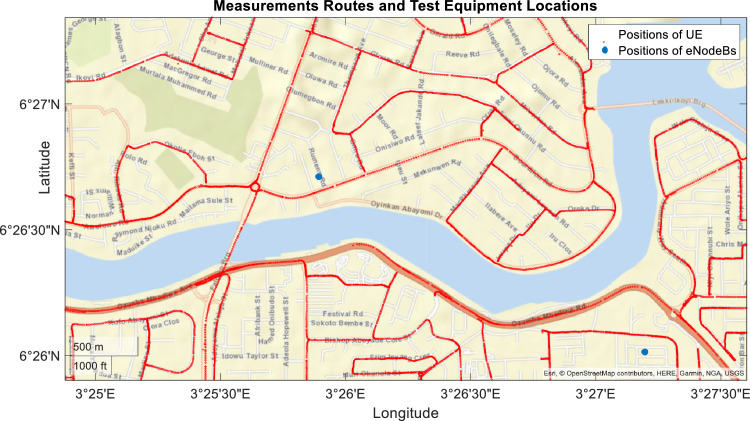
Locations of the UEs obtained from the logged longitude and latitude information.

The test vehicle was driven at 30 km/h, and field measurements were taken at 1 s intervals. The longitude and latitude information shows the route covered, as shown in Fig. 1. It is shown that the covered routes are Victoria Island (VI) and Ikoyi, Lagos, Nigeria. These are places where corporate headquarters of multinational and national companies are located. Sixteen (16) key measurements are made at one-second intervals during a 30 km/h vehicular movement of the UEs. The total duration of data measurement is 11 h, 48 min and 18 s. This time gives a total of 42,498 instances of each parameter. However, some instances returned no values. Table 1 summarises the parameters measured, the number of missing values and the summary of the existing instances, excluding the missing values.

**Table 1:** Summary of measured parameters, including the percentage of the missing KPI values.

Parameters	Number of Missing Values	Percentage of Missing Values (%)	Minimum Value	Median Value	Maximum Value
Serving RSRP	5273	12.41	-113.88	-82.44	-47.38
Serving RSRQ	5273	12.41	-21.44	-10.13	-3.25
Serving RSSI	5273	12.41	-89	-54.69	-20.44
Serving PCC SINR	5197	12.23	-20	6	35.5
Serving PCI	5273	12.41	0	251	503
Serving DL EARFCN	5273	12.41	1449	9460	9460
Serving SCC1 RSRP	20,253	47.66	-140	-82.13	-52.25
Serving SCC1 RSRQ	20,259	47.67	-25.25	-9.94	-3.5
Serving SCC1 RSSI	20,259	47.67	-81.13	-57	-28
Serving SCC1 PCI	20,253	47.66	61	282	503
Serving SCC1 SINR	15,862	37.32	-20	7	35.5
Serving SCC1 DL EARFCN	20,253	47.66	1,449	1449	9460
PCC PUSCH Power	5432	12.78	-19	17	23
PCC PUCCH Power	5406	12.72	-5	0	2
PDCP DL Throughput	6653	15.65	0	15,539	113,080
RLC DL Throughput	6653	15.65	0	15,610	110,870

### 1.2 Statistical Characteristics

The statistical characteristics of the measured data from different positions of the UEs measured at the one-second interval at 30 km/h vehicular speed are presented in Table 2, Table 3, Table 4, Table 5, Table 6, Table 7, Table 8, Table 9. Specifically, Table 2 shows the Reference Signal Received Power and Reference Signal Received Quality. In

**Table 2:** Statistics of Measured Reference Signal Received Power with 5273 missing values (12.41%) and Reference Signal Received Quality with 5273 missing values (12.41%), and their corresponding filled missing values using the PCHIP algorithm.

Statistics	RSRP Data with 5273 missing values (dBm)	RSRP Data with filled missing values (dBm)	RSRQ Data with 5273 missing values (dBm)	RSRQ Data with filled missing values (dBm)
Range	66.5	66.5	18.19	18.19
Minimum	-113.88	-113.88	-21.44	-21.44
Maximum	-47.38	-47.38	-3.25	-3.25
Median	-82.44	-83.19	-10.13	-10.38
Mean	-82.1602	-82.8240	-10.4678	-10.749
Standard Dev.	9.9896	10.0392	2.0007	2.1659
Variance	99.7915	100.7861	4.0028	4.6910
Skewness	0.0248	0.0516	-0.7610	-0.7431
Kurtosis	2.4110	2.4550	3.8688	3.6527

**Table 3:** Statistics of Measured Received Signal Strength Indicator with 5273 missing values (12.41%), and Primary Component Carrier Signal-to-Interference-and-Noise Ratio with 5197 missing values (12.23%) and their corresponding filled missing values using the PCHIP algorithm.

Statistics	RSSI Data with 5273 missing values (dBm)	RSSI Data with filled missing values (dBm)	PCC SINR Data with 5197 missing values (dB)	PCC SINR Data with filled missing values (dB)
Range	68.56	68.56	55.5	55.5
Minimum	-89	-89	-20	-20
Maximum	-20.44	-20.44	35.5	35.5
Median	-54.69	-55.13	6	5
Mean	-54.2650	-54.6353	6.7037	5.8991
Standard Dev.	8.9009	8.8919	6.6588	6.8153
Variance	79.2265	79.0656	44.3393	46.4489
Skewness	0.1035	0.1077	0.3262	0.3473
Kurtosis	2.4732	2.5828	2.7399	2.7946

**Table 4:** Statistics of Measured Physical Cell Identity with 5273 missing values (12.41%) and Downlink E-UTRA Absolute Radio Frequency Channel Number with 5273 missing values (12.41%) and their corresponding filled missing values using the PCHIP algorithm.

Statistics	PCI Data with 5273 missing values	PCI Data with filled missing values	DL EARFCN Data with 5273 missing values	DL EARFCN Data with filled missing values
Range	503	503	8011	8011
Minimum	0	0	1449	1449
Maximum	503	503	9460	9460
Median	251	250.3968	9460	9460
Mean	257.2823	257.1113	8,935.7	8825.9
Standard Dev.	127.9588	126.9824	1,980.3	2142.8
Variance	16,373	16,125	3,921,800	4,591,600
Skewness	0.1663	0.1601	-3.5148	-3.1166
Kurtosis	1.7277	1.7390	13.3568	10.7634

**Table 5:** Statistics of Measured 1st Secondary Component Carrier RSRP with 20253 missing values (47.66%) and SCC1 RSRQ with 20259 missing values (47.67%) and their corresponding filled missing values using the PCHIP algorithm.

Statistics	SCC1 RSRP Data with 20253 missing values (dBm)	SCC1 RSRP Data with filled missing values (dBm)	SCC1 RSRQ Data with 20259 missing values (dBm)	SCC1 RSRQ Data with filled missing values (dBm)
Range	87.75	50,725	21.75	9,354.8
Minimum	-140	-50,778	-25.25	-25.25
Maximum	-52.25	-52.25	-3.5	9329.6
Median	-82.13	-84.88	-9.94	-10.6709
Mean	-82.3437	-95.8292	-10.5370	-9.1679
Standard Dev.	9.5658	577.0000	2.4865	105.9448
Variance	91.5048	332,930	6.1827	11,224
Skewness	-0.1570	-64.0260	-1.4785	64.2687
Kurtosis	2.8304	4,504.8	6.1366	4,539.7

**Table 6:** Statistics of Measured SCC1 RSSI with 20259 missing values (47.67%) and SCC1 PCI with 20253 missing values (47.66%) and their corresponding filled missing values using the PCHIP algorithm.

Statistics	SCC1 RSSI Data with 20259 missing values (dBm)	SCC1 RSSI Data with filled missing values (dBm)	SCC1 PCI Data with 20253 missing values	SCC1 PCI Data with filled missing values
Range	53.13	69,535	442	442
Minimum	-81.13	-69,563	61	61
Maximum	-28	-28	503	503
Median	-57	-58.7467	282	256.5306
Mean	-56.9909	-73.4214	277.9448	266.5715
Standard Dev.	8.4770	790.9646	132.7527	128.0515
Variance	71.8601	625,620	17,623	16,397
Skewness	0.0319	-64.0543	-0.0113	0.1045
Kurtosis	2.4225	4,507.8	1.7525	1.7951

**Table 7:** Statistics of Measured SCC1 SINR with 15862 missing values (37.32%) and the corresponding filled missing values using the PCHIP algorithm.

Statistics	SCC1 SINR Data with 15862 missing values (dB)	SCC1 SINR Data with filled missing values (dB)	SCC1 DL EARFCN Data with 20253 missing values	SCC1 DL EARFCN Data with filled missing values
Range	55.5	55.5	8011	8011
Minimum	-20	-20	1449	1449
Maximum	35.5	35.5	9460	9460
Median	7	4.6606	1449	1449
Mean	7.4047	5.0454	1603.1	1901.4
Standard Dev.	8.0043	8.3551	1100.5	1695.2
Variance	64.0684	69.8076	1,211,110	2,873,700
Skewness	-0.0137	0.0422	6.9996	3.7938
Kurtosis	3.1080	3.0712	49.9939	15.9998

**Table 8:** Statistics of Measured PCC Physical Uplink Shared Channel with 5432 missing values (12.78%) and PCC Physical Uplink Control Channel Power with 5406 missing values (12.72%) and their corresponding filled missing values using the PCHIP algorithm.

Statistics	PCC PUSCH Data with 5432 missing values (dBm)	PCC PUSCH Data with filled missing values (dBm)	PCC PUCCH Data with 5406 missing values (dBm)	PCC PUCCH Data with filled missing values (dBm)
Range	42	42	7	7
Minimum	-19	-19	-5	-5
Maximum	23	23	2	2
Median	17	17	0	0
Mean	15.5391	15.8916	-0.34	-0.2804
Standard Dev.	6.6191	6.5029	1.2546	1.2446
Variance	43.8120	42.2878	1.5741	1.5489
Skewness	-1.2164	-1.2734	-0.4249	-0.4343
Kurtosis	4.5527	4.7315	3.2037	3.2538

**Table 9:** Statistics of Measured Packet Data Convergence Protocol Throughput for the Downlink with 6653 missing values (15.65%) and Radio Link Control Throughput for the Downlink with 6653 missing values (15.65%) and their corresponding filled missing values using the PCHIP algorithm.

Statistics	PDCP Throughput DL Data with 6653 missing values (bps)	PDCP Throughput DL Data with filled missing values (bps)	RLC Throughput DL Data with 6653 missing values (bps)	RLC Throughput DL Data with filled missing values (bps)
Range	113,080	177,840	110,872	178,400
Minimum	0	-64,759	0	-67,524
Maximum	113,080	113,080	110,872	110,872
Median	15,539	13,278	15,610	13,321
Mean	22,389	20,328	22,445	20,378
Standard Dev.	21,102	20,451	21,137	20,488
Variance	445,300,000	418,220,000	419,760,000	419,760,000
Skewness	1.4078	1.5345	1.4058	1.5329
Kurtosis	4.7811	5.2706	4.7716	5.2628

Table 3, the Received Signal Strength Indicator and Primary Component Carrier Signal-to-Interference-and-Noise Ratio are highlighted. Table 4 shows the Physical Cell Identity and E-UTRA Absolute Radio Frequency Channel Number. The 1st Secondary Component Carrier RSRP and SCC1 RSRQ are shown in Table 5. In addition, Table 6 shows the SCC1 RSSI and SCC1 PCI. Table 7 presents SCC1 SINR and SCC1 DL EARFCN. The PCC Physical Uplink Shared Channel Power and PCC Physical Uplink Control Channel Power are given in Table 8. Finally, the Packet Data Convergence Protocol Throughput for the Downlink and Radio Link Control Throughput DL are shown in Table 9.

### 1.3 Explanation of the Piecewise Cubic Hermite Interpolating Polynomial (PCHIP)

The PCHIP is a distinctive third-degree piecewise polynomial function with robust shape-preserving characteristics than cubic splines. The peculiar robust shape-preserving feature makes the PCHIP an attractive technique for detailed dataset curve fitting and analysis in this paper.

Typically, the PCHIP is suitable for interpolating numeric datasets with specified intervals and values $x_{0},x_{1},x_{2},\ldots ,x_{n}$ to attain a function. Supposing a function f(x) containing $x_{0},x_{1}$ has a fourth-order derivative. It is defined within the interval where [p,q], For any x∈[p,q],there is always ε∈(p,q),(εdependonx). Then employing the following interpolation conditions given by Eqs. (1) and (2). The accuracy of the PCHIP algorithm is then analyzed using statistical error analysis. These analyses could also be further enhanced by investigating the outliers of the processed, filled missing values and either exempting those values from the investigations or further interpolating those outlier values. A detailed description of the PCHIP algorithm and the associated equations is provided in the following literature [5], [6], [7], [8].

(1) $$H_{3}\left(x_{o}\right)=H_{3^{\prime }}\left(x_{o}\right)=0,\;\;H_{3}\left(x_{1}\right)=H_{3^{\prime }}\left(x_{1}\right)=0.$$

(2) $$H_{3}\left(x\right)=C\left(x\right){\left(x-x_{o}\right)}^{2}{\left(x-x_{1}\right)}^{2}$$

In data analysis, the application of cubic splines to interpolate a time series can result in unrealistic overshoots, which is undesirable in practice. More often, when there is an increment in the independent variable resulting in remarkable variations between successive samples, unrealistic overshoots occur. In order to address this problem, we employed the PCHIP, which can overcome unrealistic overshoots. The data points strictly bound the PCHIP interpolant by its design. Here, the cubic polynomial between a pair of tested data points in the PCHIP algorithm is derived using the data values at these points and the selected values of the derivatives at the specified data points. A critical examination of the specific data point aids in choosing the value of the derivative at a given data point and the data point to its left and right. In summary, our justification for using the PCHIP method lies in its capability to deal with unrealistic overshoots.

### 1.4 Statistical Error Analyses

The Piecewise Cubic Hermite Interpolating Polynomial (PCHIP) algorithm was used in predicting the missing values for each parameter. Here, the statistical error with missing values exempted and with missing values filled with the PCHIP algorithm are compared. The statistical errors of interest are the Mean Absolute Error (MAE), Root Mean Square Error (RMSE), Relative Absolute Error (RAE) and Mean Squared Error (MSE) [19,20]. Table 10 shows the MAE and the RMSE analyses, and Table 11 shows the RAE and the MSE analyses. It can be seen that predicting/estimating the missing values for the measured parameters using the PCHIP algorithm is accurate as the errors are not worse than when the missing values are exempted from the analyses except in the cases of PCC PUSCH power and PCC PUCCH power.

**Table 10:** Results showing the Mean Absolute Error (MAE) and Root Mean Square Error (RMSE) between the data (histograms) and the corresponding probability density function (pdf) curves for both the measured data with missing values and the data where the missing data are filled using the PCHIP algorithm.

Statistics	MAE with missing data	MAE with filled missing data	RMSE with missing data	RMSE with filled missing data
RSRP	0.0051	0.0050	0.0065	0.0067
RSRQ	0.0122	0.0134	0.0192	0.0203
RSSI	0.0043	0.0042	0.0062	0.0063
PCC SINR	0.0033	0.0032	0.0051	0.0050
PCI	0.0013	0.0013	0.0016	0.0017
DL EARFCN	0.0002	0.0002	0.0006	0.0007
SCC1 RSRP	0.0060	0.0000	0.0095	0.0006
SCC1 RSRQ	0.0236	0.0001	0.0439	0.0022
SCC1 RSSI	0.0097	0.0000	0.0128	0.0004
SCC1 PCI	0.0010	0.0012	0.0013	0.0016
SCC1 SINR	0.0057	0.0024	0.0088	0.0041
SCC1 DL EARFCN	0.0001	0.0002	0.0005	0.0007
PCC PUSCH	0.0095	0.0216	0.0213	0.0469
PCC PUCCH	0.0362	0.2283	0.0516	0.5460
PDCP Th. DL	0.0000	0.0000	0.0000	0.0000
RLC Th. DL	0.0000	0.0000	0.0000	0.0000

**Table 11:** Results showing the Relative Absolute Error (RAE) and Mean Squared Error (MSE) between the data (histograms) and the corresponding probability density function (pdf) curves for both the measured data with missing values and the data where the missing data are filled using the PCHIP algorithm.

Statistics	RAE with missing data	RAE with filled missing data	MSE with missing data	MSE with filled missing data
RSRP	0.3694	0.3331	0.0000	0.0000
RSRQ	0.2366	0.2292	0.0004	0.0004
RSSI	0.3368	0.2960	0.0000	0.0000
PCC SINR	0.2093	0.1834	0.0000	0.0000
PCI	0.6921	0.6571	0.0000	0.0000
DL EARFCN	0.9722	0.9759	0.0000	0.0000
SCC1 RSRP	1.0000	0.9683	0.0001	0.0000
SCC1 RSRQ	1.0000	0.9408	0.0019	0.0000
SCC1 RSSI	1.0000	0.9584	0.0002	0.0000
SCC1 PCI	0.9998	0.6119	0.0000	0.0000
SCC1 SINR	0.5523	0.1654	0.0000	0.0000
SCC1 DL EARFCN	0.9549	0.9605	0.0000	0.0000
PCC PUSCH	0.6041	0.8183	0.0005	0.0022
PCC PUCCH	0.3271	0.9475	0.0027	0.2981
PDCP Th. DL	1.3708	1.1731	0.0000	0.0000
RLC Th. DL	1.3448	1.1503	0.0000	0.0000

### 1.5 Probability Distribution

In order to emphasize the importance of data preprocessing in 4G LTE data analysis, the normalized histograms of the original measured data with missing values and the histograms when all the missing data are filled are plotted. The probability density function is used for the normalization [21]. After that, the distribution of the histogram is examined, and the corresponding probability density function (pdf) curves for the distribution are drawn for both the measured initially data with missing values and the corresponding filled data of the same parameters. For instance, the pdf for normal distribution is given in Eq. (3). Similarly, the pdf for half-normal distribution is shown in Eq. (4).

(3) $$pdf\left(x,\mu ,\sigma \right)=\frac{1}{\sigma \sqrt{2\pi }}e^{\left[\frac{-{\left(x-\mu \right)}^{2}}{2\sigma^{2}}\right]}$$

(4) $$pdf\left(x,\mu ,\sigma \right)=\sqrt{\frac{2}{\pi }}\frac{1}{\sigma }e^{-1\;/\;2{\left(\frac{x-\mu }{\sigma }\right)}^{2}}$$

where σ is the standard deviation, μ is the mean, and $\sigma^{2}$ describes the variance.

Fig. 18, Fig. 19, Fig. 20, Fig. 21, Fig. 22, Fig. 23, Fig. 24, Fig. 25, Fig. 26 use the pdf for normal distribution, Eq. (3), to plot the pdf curves, while Figs. 27 and 28 use the half-normal equation, Eq. (4), to plot the pdf curves. The pdf-normalized data exempting the missing values and filling the missing values with the PCHIP algorithm are as shown in the histograms. Specifically, Figs. 18, Fig. 19 and Fig. 20 show the histograms and pdf curves for the RSRP, RSRQ and RSSI data, respectively. The PCC SINR and PCI data are shown in the histograms and pdf curves of Fig. 21 and Fig. 22, respectively. Figs. 23 and 24 show the SCC1 PCI and SCC1 SINR, respectively. The PCC PUSCH power and PCC PUCCH power data are presented graphically in Fig. 25 and Fig. 26, respectively. Finally, Fig. 27 and Fig. 28 show graphical representations of PDCP Throughput DL and RLC Throughput DL, respectively.

**Fig. 2:**
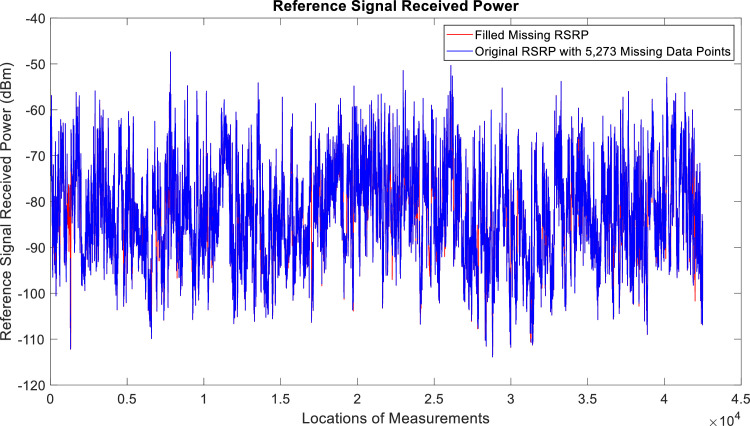
Reference Signal Received Power obtained from locations of the UEs.

**Fig. 3:**
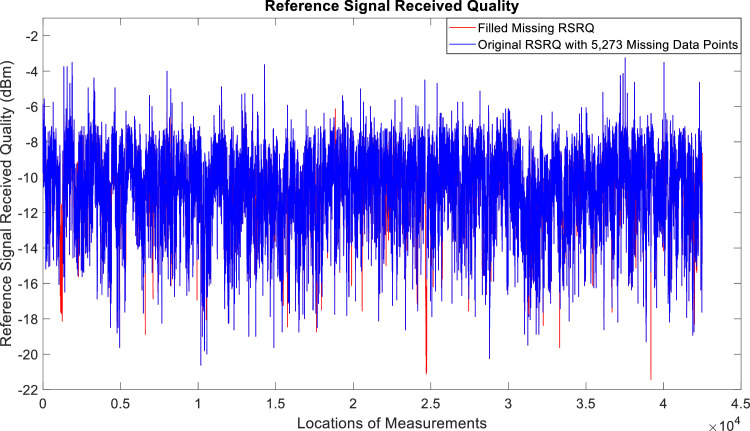
Reference Signal Received Quality measurements obtained from locations of the UEs.

**Fig. 4:**
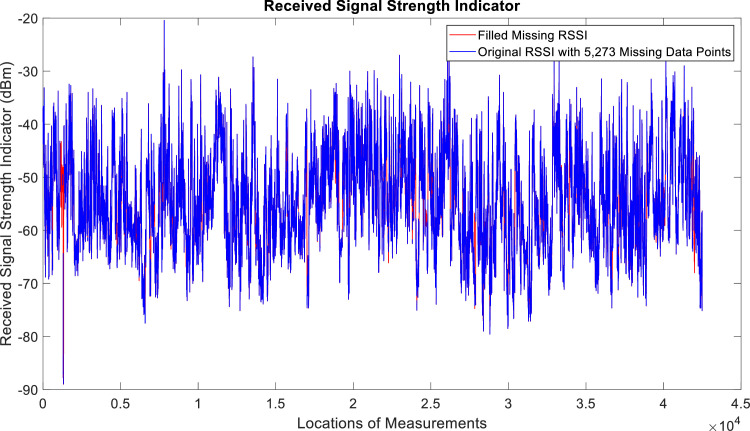
Received Signal Strength Indicator obtained from locations of the UEs.

**Fig. 5:**
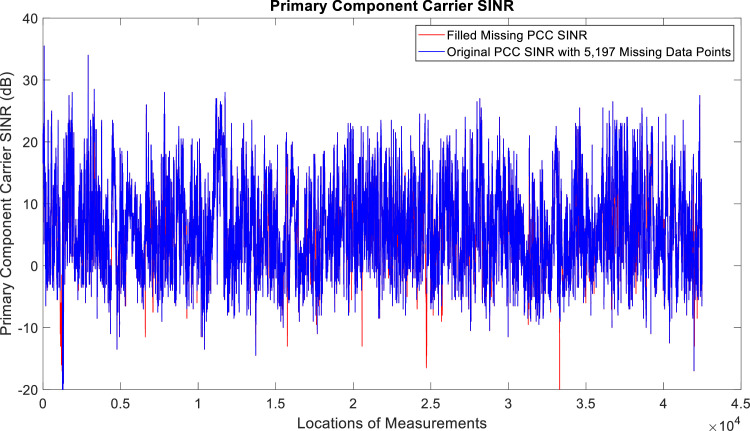
Primary Component Carrier Signal-to-Interference-and-Noise Ratio obtained from locations of the UEs.

**Fig. 6:**
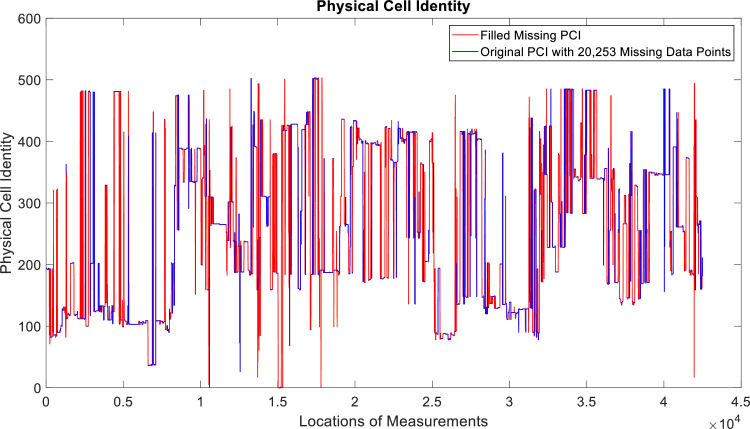
Physical Cell Identity obtained from locations of the UEs.

**Fig. 7:**
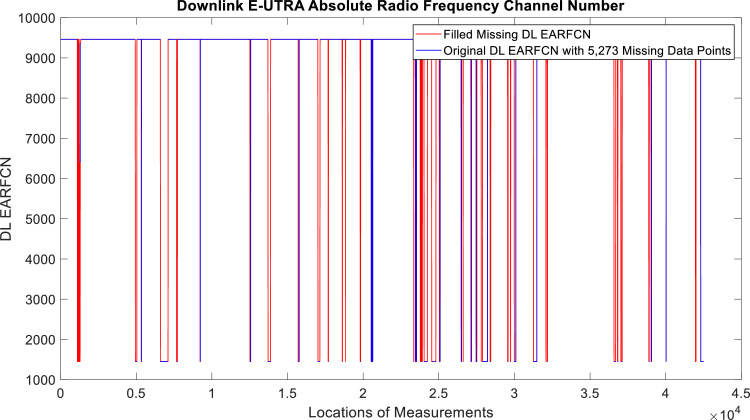
Downlink E-UTRA Absolute Radio Frequency Channel Number obtained from locations of the UEs.

**Fig. 8:**
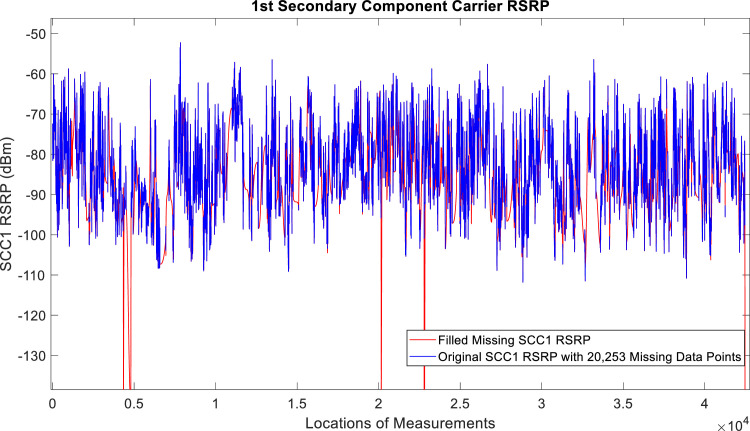
1st Secondary Component Carrier RSRP obtained from locations of the UEs.

**Fig. 9:**
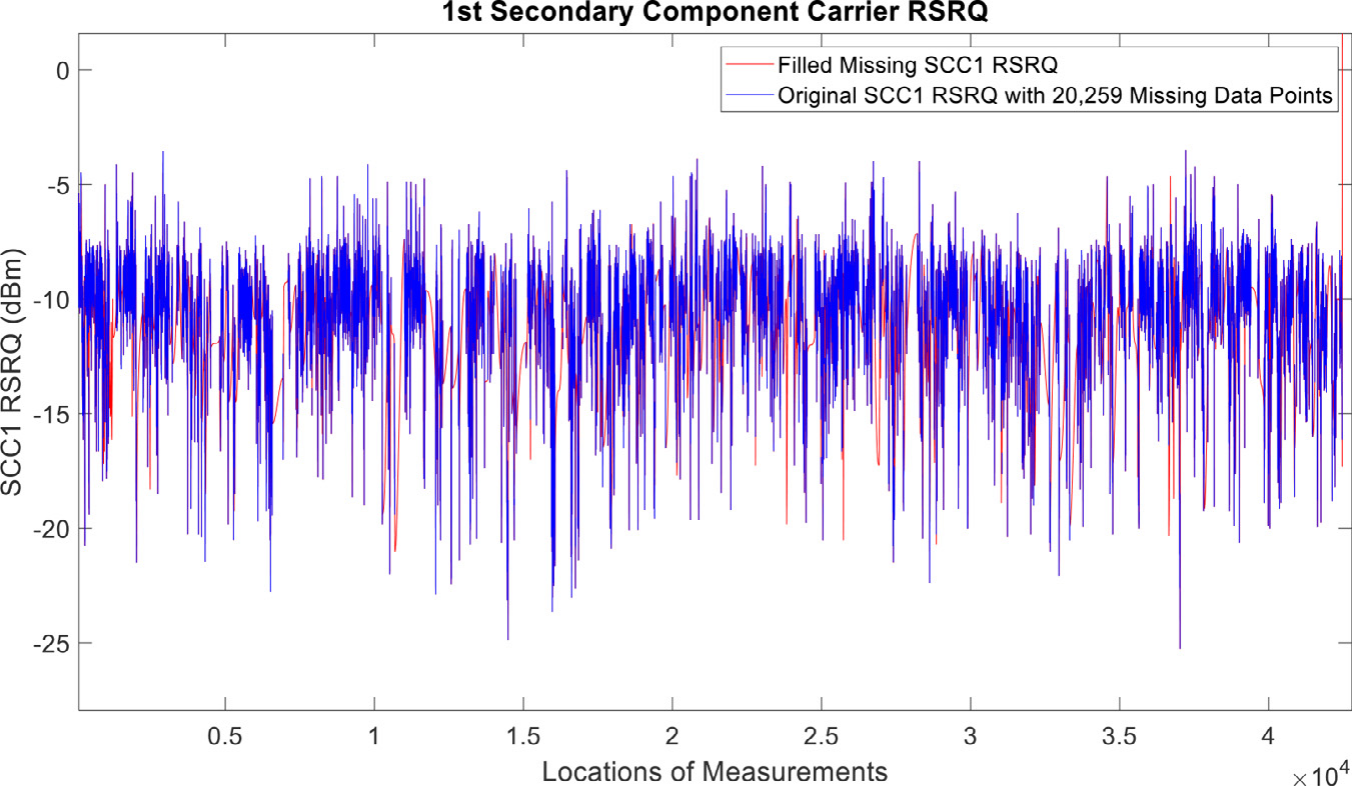
1st Secondary Component Carrier RSRQ obtained from locations of the UEs.

**Fig. 10:**
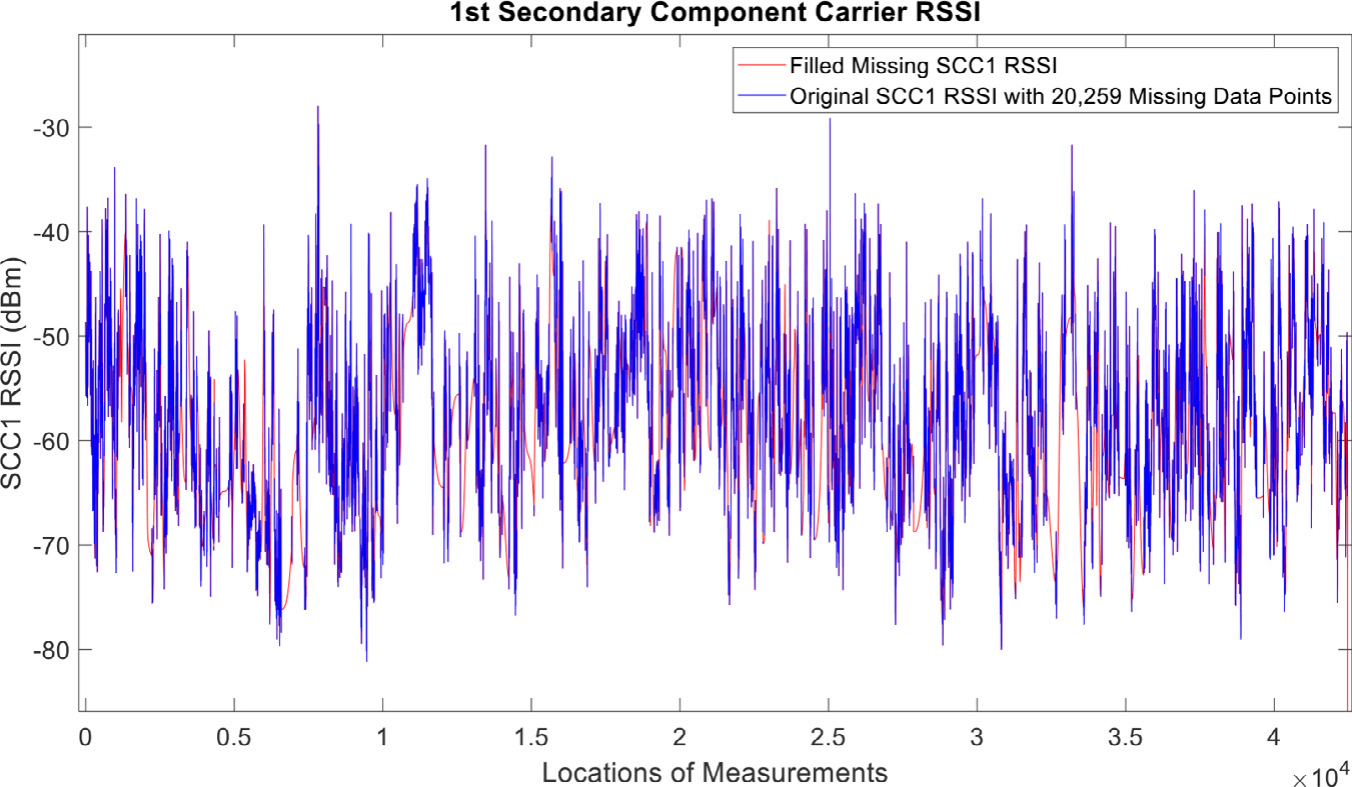
1st Secondary Component Carrier RSSI obtained from locations of the UEs.

**Fig. 11:**
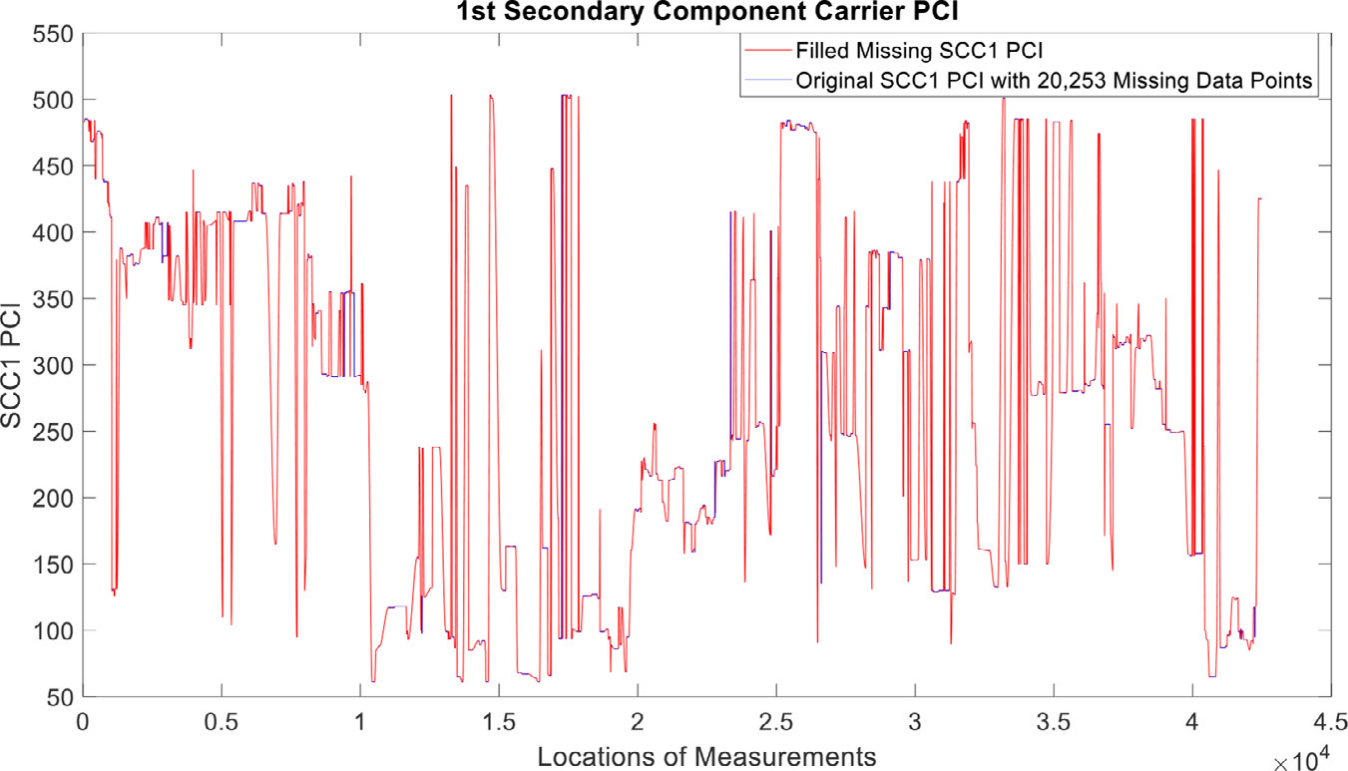
1st Secondary Component Carrier PCI obtained from locations of the UEs.

**Fig. 12:**
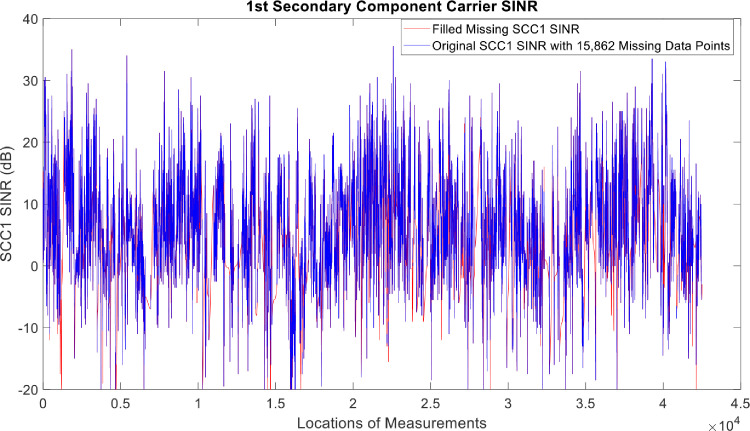
1st Secondary Component Carrier SINR obtained from locations of the UEs.

**Fig. 13:**
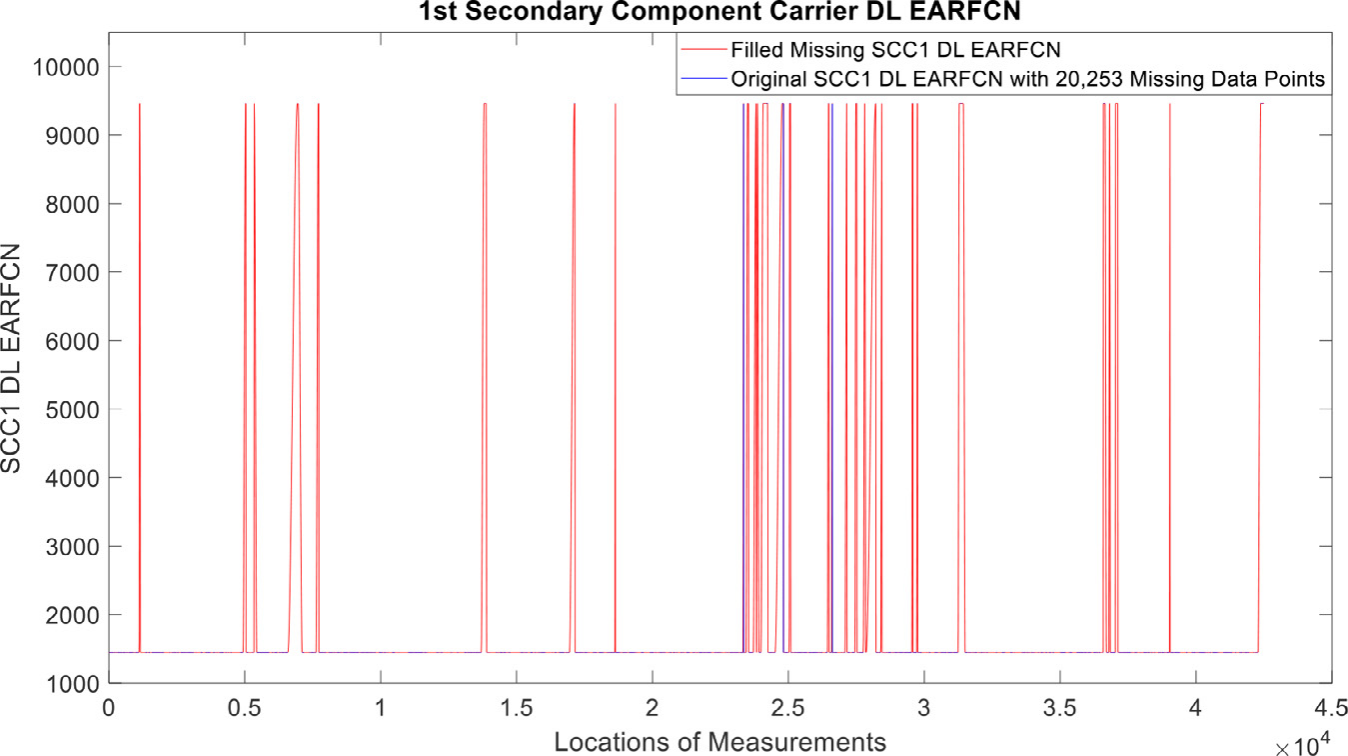
1st Secondary Component Carrier DL EARFCN obtained from locations of the UEs.

**Fig. 14:**
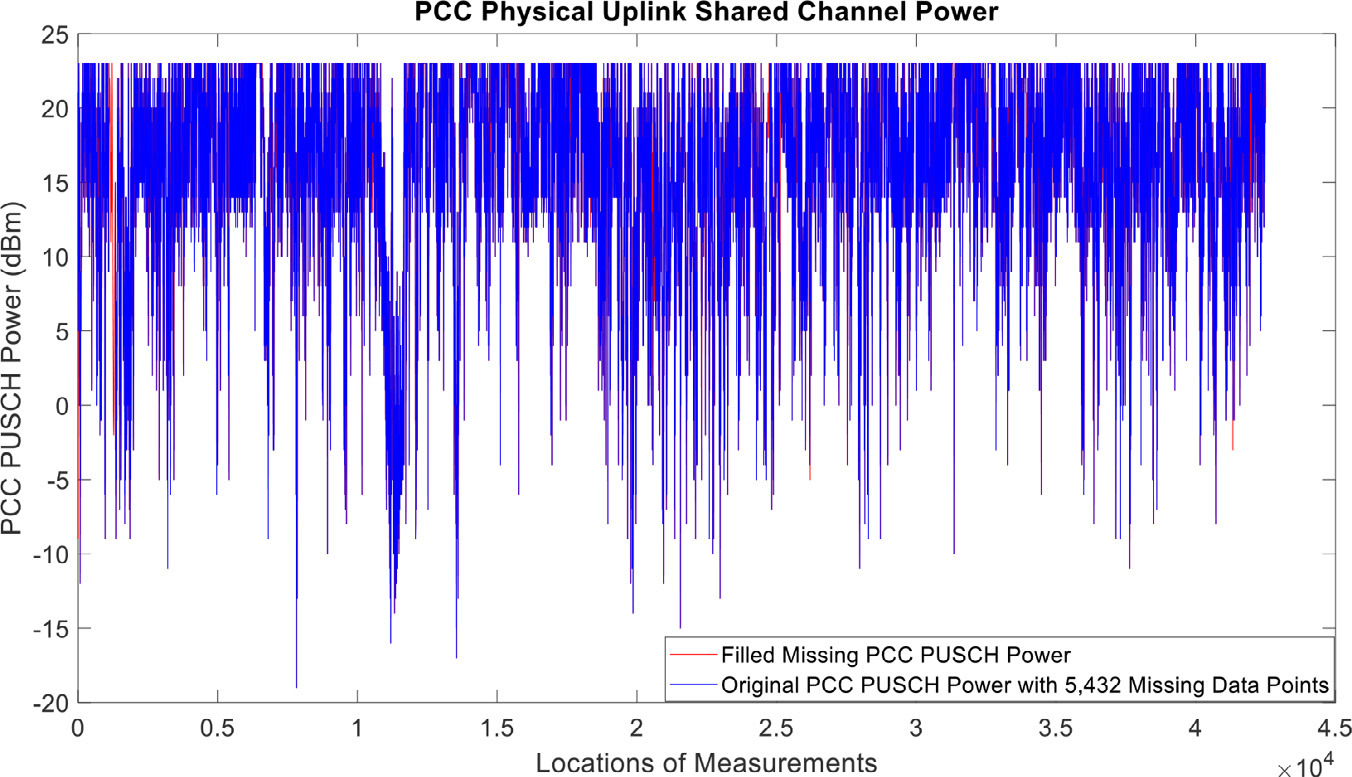
Primary Component Carrier Physical Uplink Shared Channel Power obtained from locations of the UEs.

**Fig. 15:**
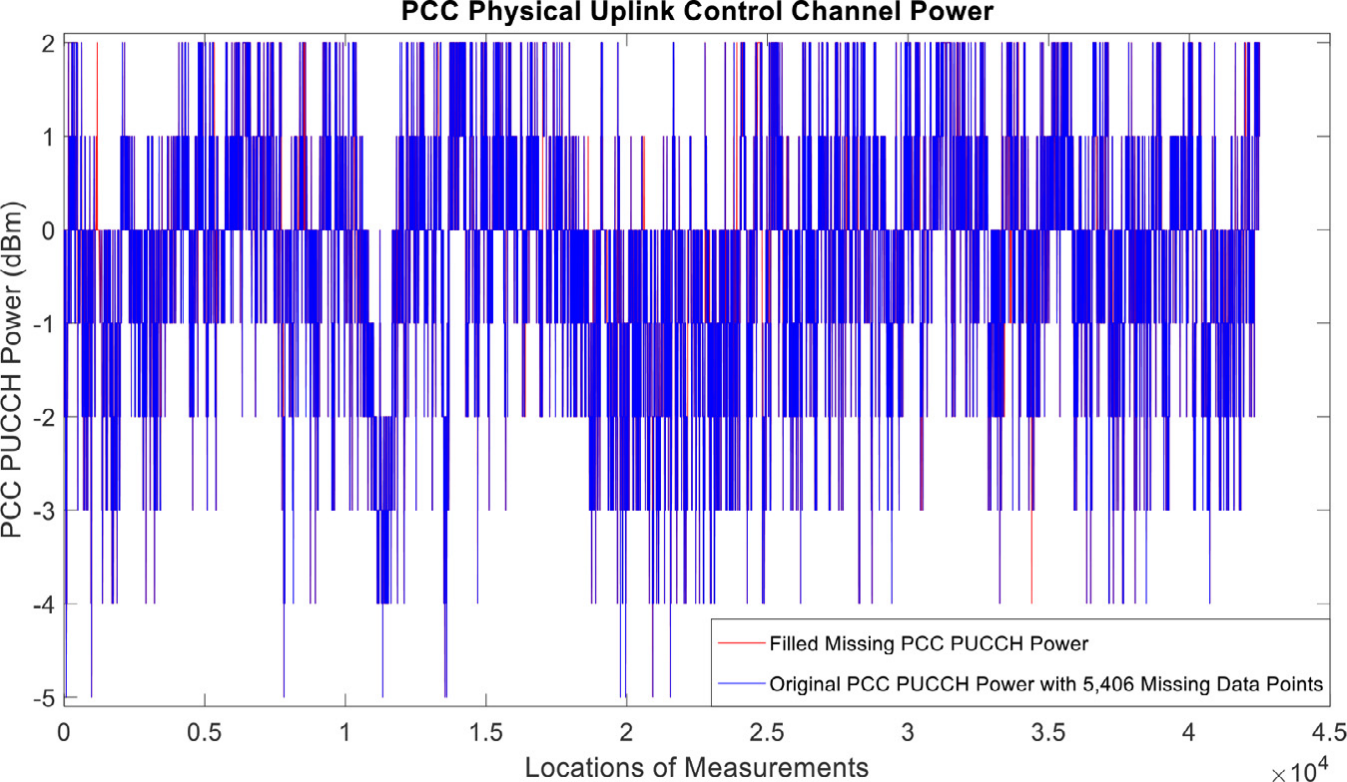
PCC Physical Uplink Control Channel Power obtained from locations of the UEs.

**Fig. 16:**
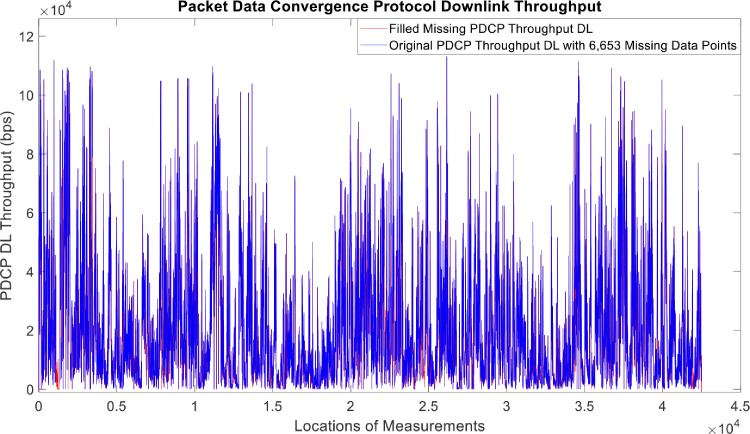
Packet Data Convergence Protocol Downlink Throughput obtained from locations of the UEs.

**Fig. 17:**
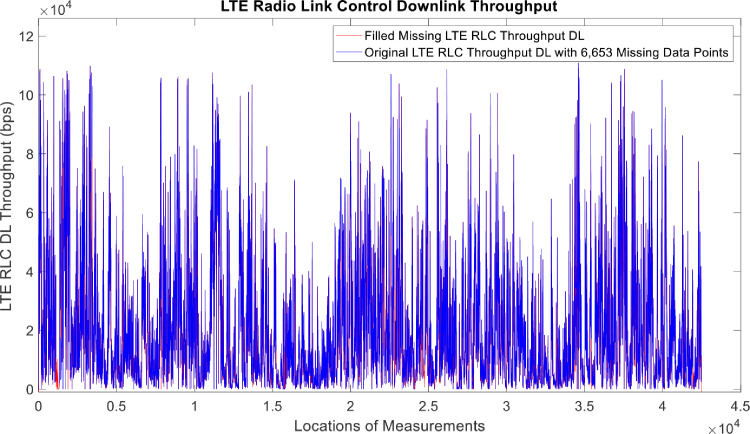
Radio Link Control Downlink Throughput obtained from locations of the UEs.

Fig. 2, Fig. 3, Fig. 4, Fig. 5, Fig. 6, Fig. 7, Fig. 8, Fig. 9, Fig. 10, Fig. 11, Fig. 12, Fig. 13, Fig. 14, Fig. 15, Fig. 16, Fig. 17 show the measured data from the UEs’ locations. The missing instances of each parameter are filled by leveraging the Piecewise Cubic Hermite Interpolating Polynomial (PCHIP) algorithm. Specifically, Fig. 2, Fig. 3, and Fig. 4 present the serving Reference Signal Received Power, Reference Signal Received Quality and Reference Signal Strength Indicator, respectively. Also, Fig. 5, Fig. 6, and Fig. 7 present the serving Primary Component Carrier Signal-to-Interference-and-Noise Ratio, Physical Cell Identity, and the Downlink E-UTRA Absolute Radio Frequency Channel Number, respectively. Figs. 8 to 13 are the serving 1st Secondary Component Carrier RSRP, RSRQ, RSSI, PCI, SINR and DL EARFCN. Fig. 14 and Fig. 15 present the Primary Component Carrier Physical Uplink Shared Channel Power and Physical Uplink Control Channel Power, respectively. In addition, Fig. 16 represents the Packet Data Convergence Protocol for the Downlink. Finally, Fig. 17 illustrates the Radio Link Control Throughput for the Downlink.

**Fig. 18:**
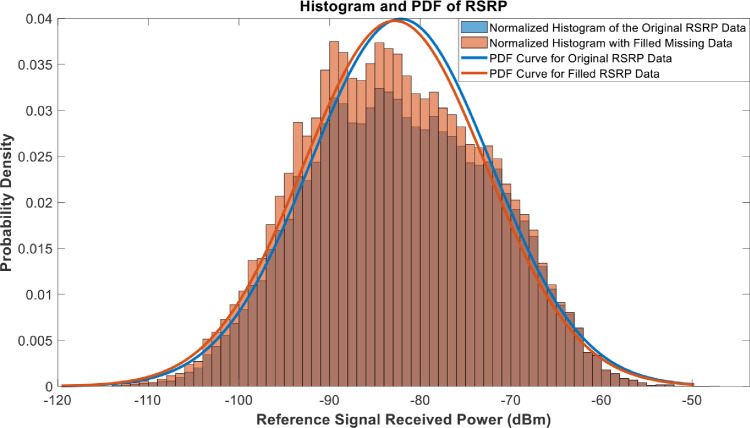
Histograms and PDF curves of RSRP of measured data with missing values and filled values.

**Fig. 19:**
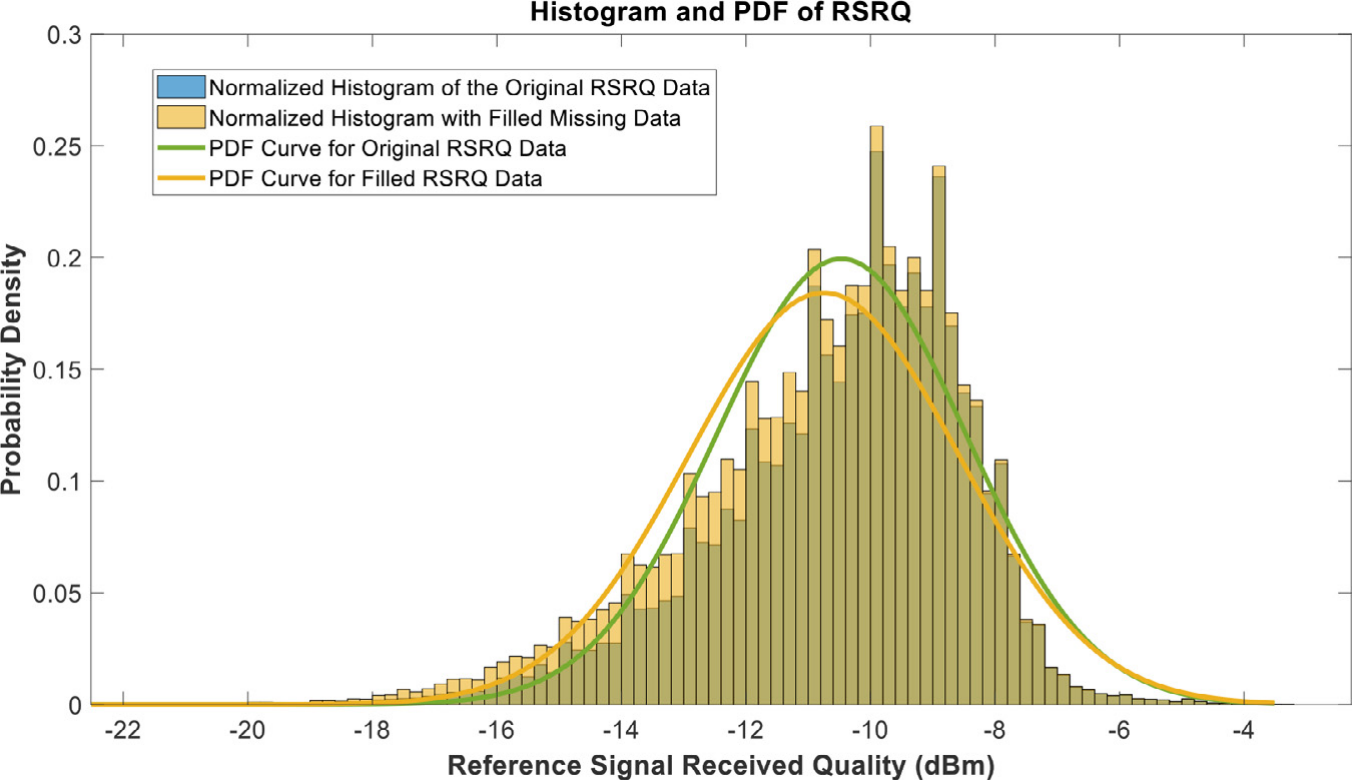
Histograms and PDF curves of RSRQ of measured data with missing values and filled values.

**Fig. 20:**
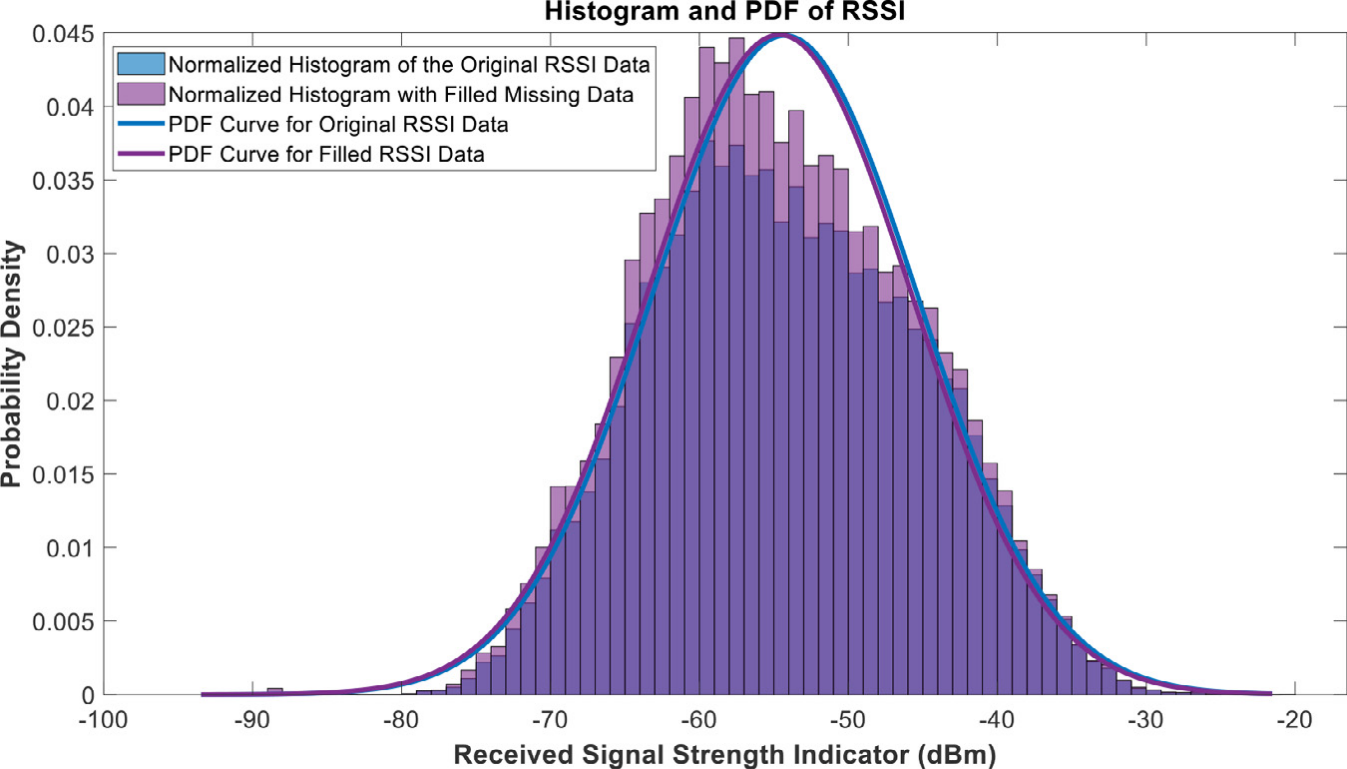
Histograms and PDF curves of RSSI of measured data with missing values and filled values.

**Fig. 21:**
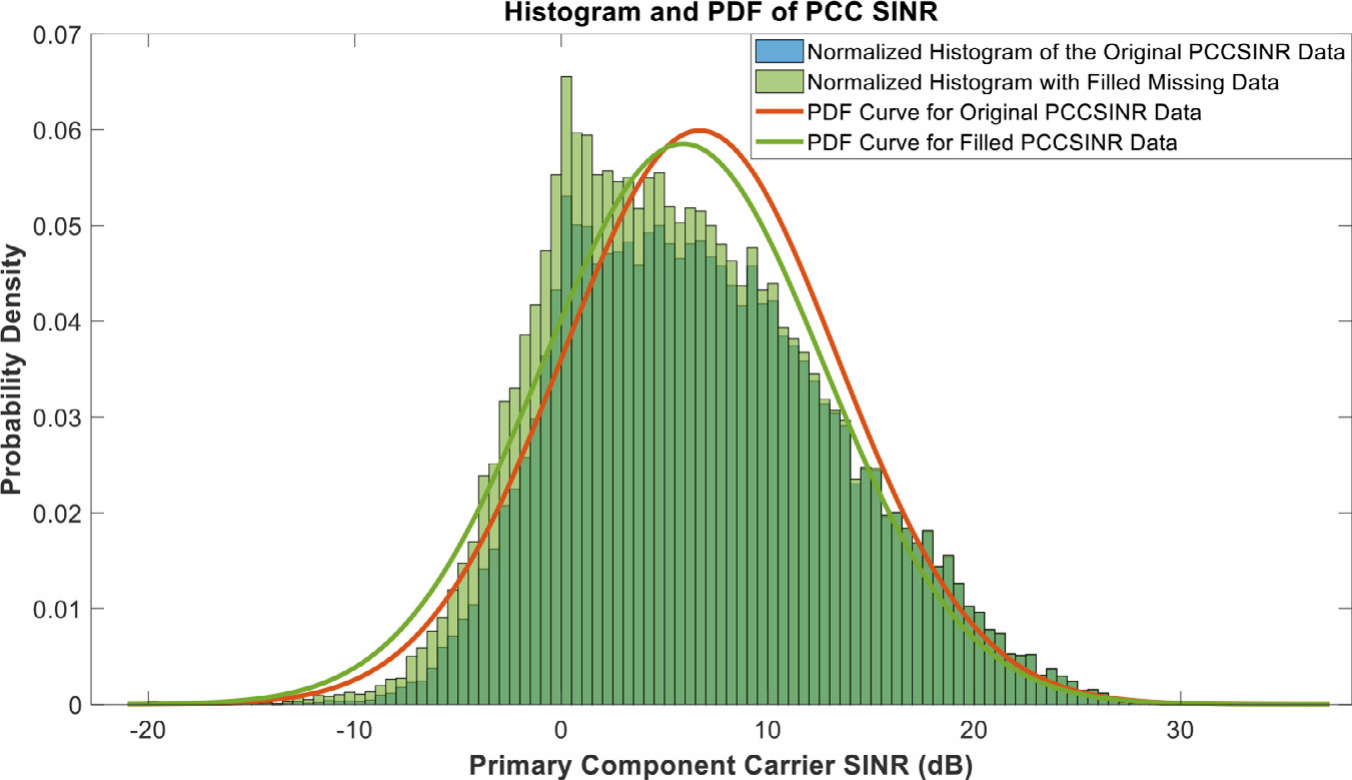
Histograms and PDF curves of PCC SINR of measured data with missing values and filled values.

**Fig. 22:**
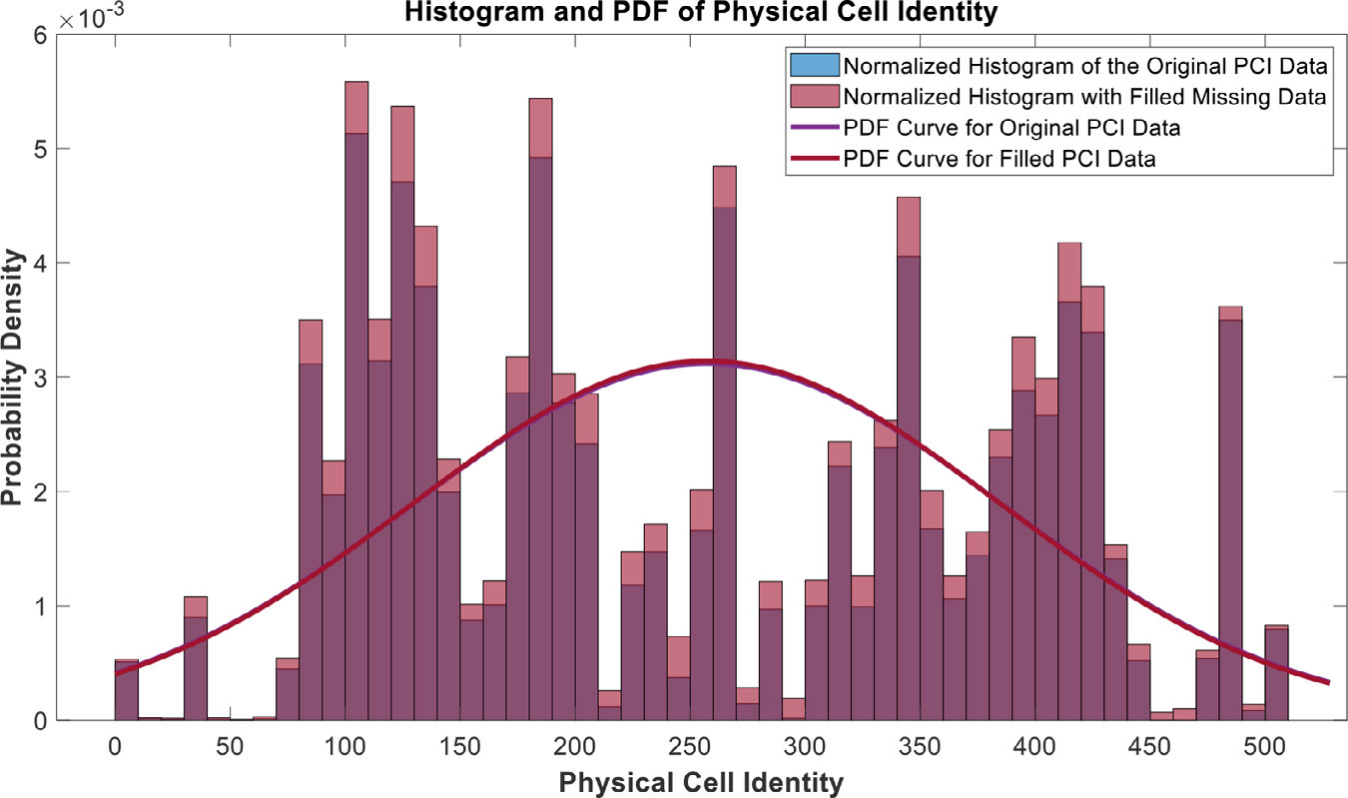
Histograms and PDF curves of PCI of measured data with missing values and filled values.

**Fig. 23:**
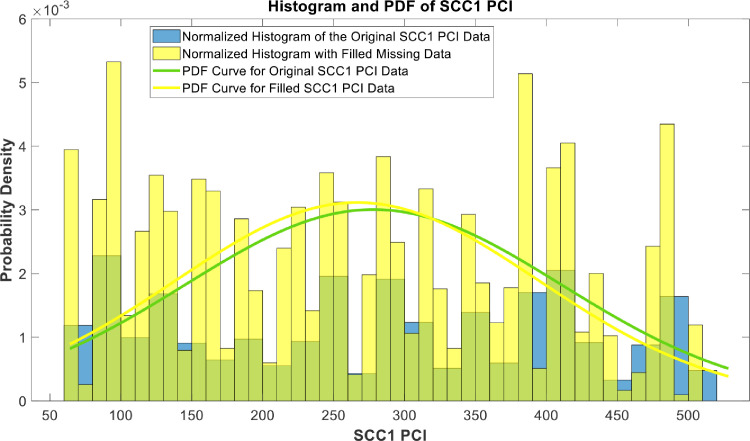
Histograms and PDF curves of SCC1 PCI of measured data with missing values and filled values.

**Fig. 24:**
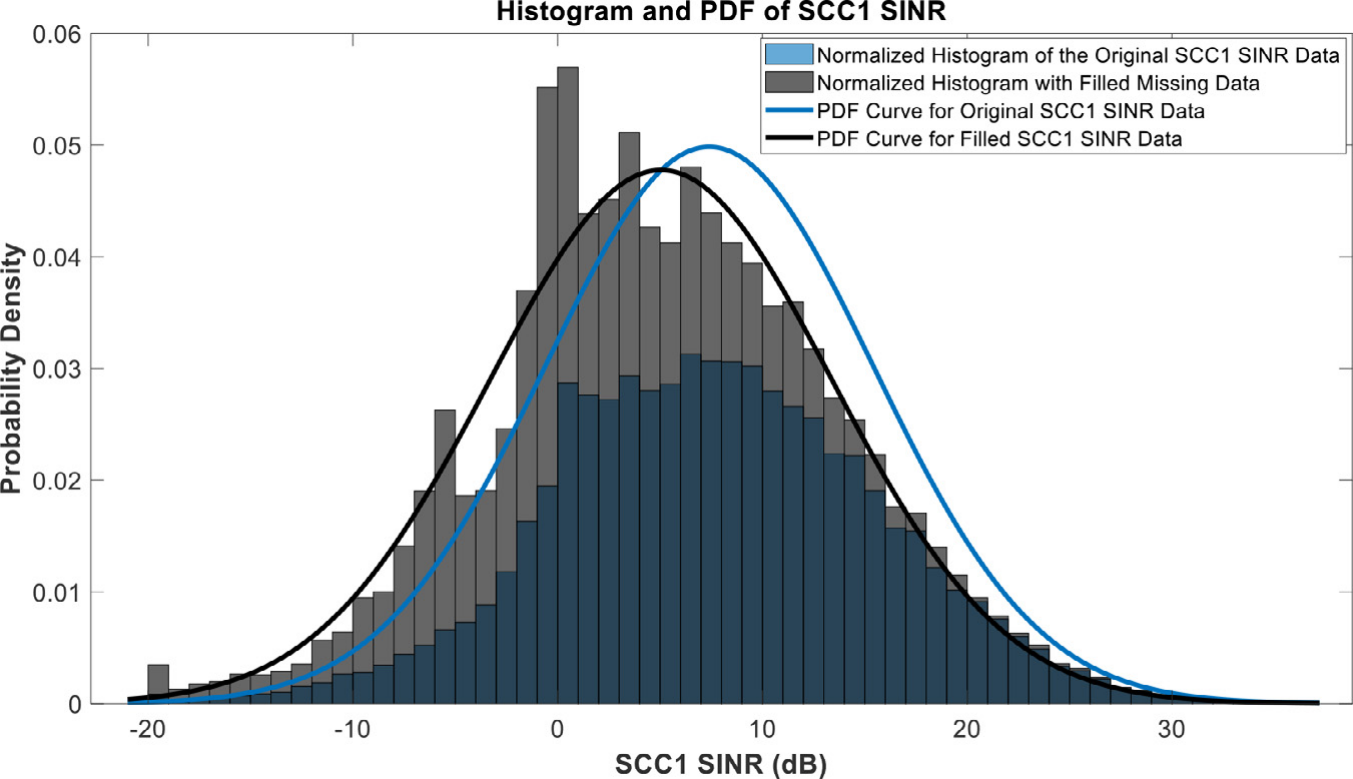
Histograms and PDF curves of SCC1 SINR of measured data with missing values and filled values.

**Fig. 25:**
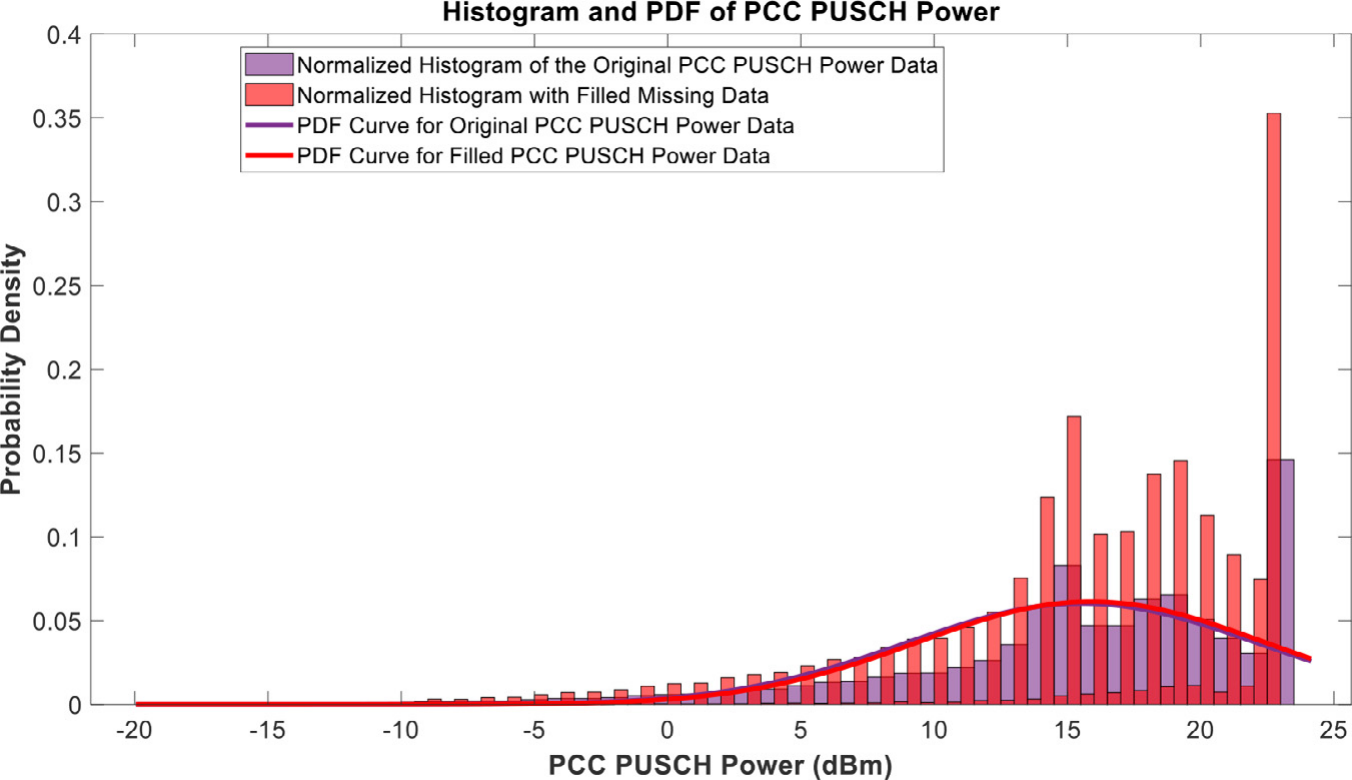
Histograms and PDF curves of PCC PUSCH of measured data with missing values and filled values.

**Fig. 26:**
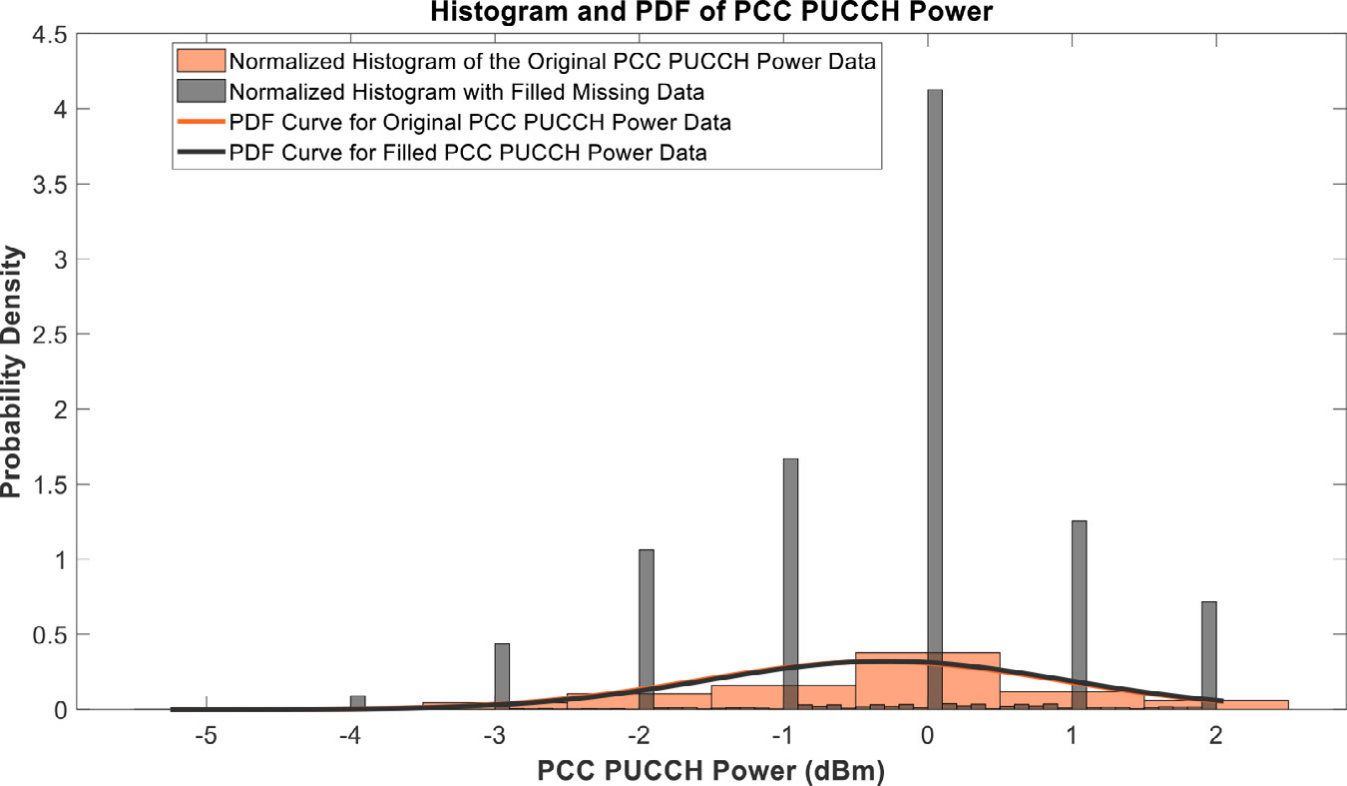
Histograms and PDF curves of PCC PUCCH of measured data with missing values and filled values.

**Fig. 27:**
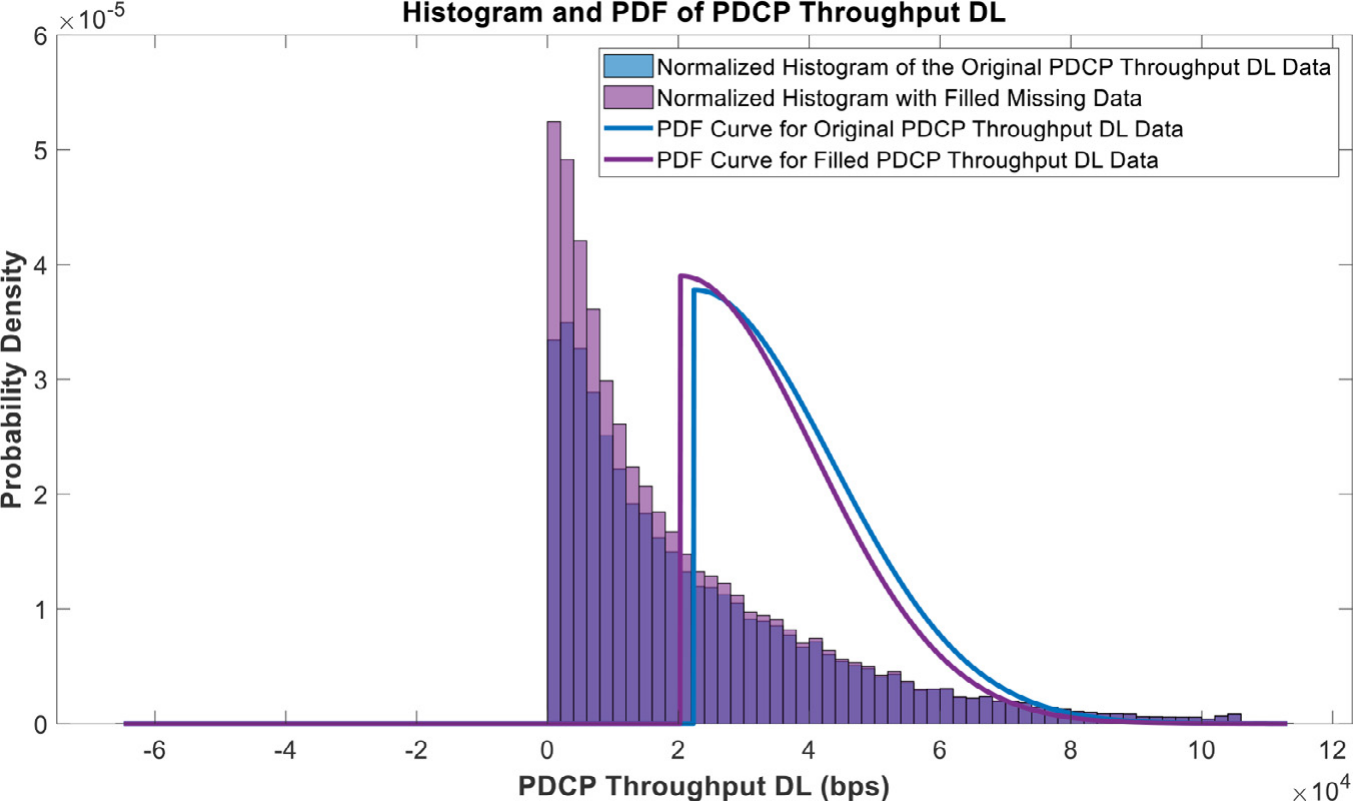
Histograms and PDF curves of PDCP Throughphput for the Downlink of measured data with missing values and filled values.

**Fig. 28:**
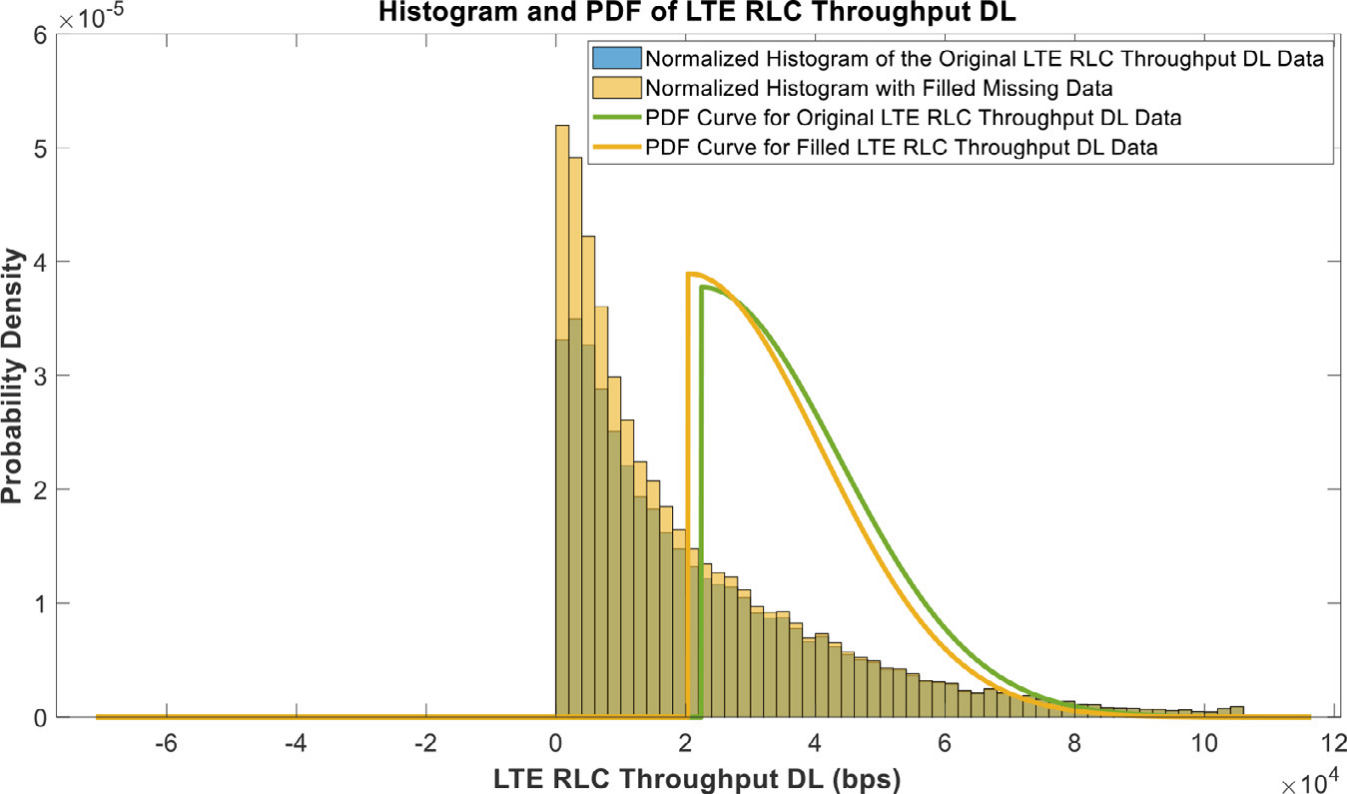
Histograms and PDF curves of LTE RLC Throughput for the Downlink of measured data with missing values and filled values.

## 2 Experimental Design, Materials and Methods

A 4G LTE modem was used in acquiring the field data. The device, equipped with a 2×2 MIMO antenna with 64 Quadrature Amplitude Modulation (QAM) capability, is mounted on a vehicle driven at an approximately constant speed of 30 km/h. The data are logged at the 1 s interval and time-stamped. Thirty-two parameters, including the logging time, longitude and latitude, were recorded every second. Data were logged for a total number of forty-two thousand, four hundred and ninety-eight instances. In particular, this corresponds to 11 h, 48 min and 18 s measurement duration of logging data every second. However, some cases were not logged for each parameter due to severe path losses caused by huge separation distances between transmitters and the receivers and other obstructions in the line of sight. We use MATLAB R2020a installed on a personal computer for data curation and analysis. In particular, the data were analyzed with the exemption of the missing values. After that, the missing values were estimated using the Piecewise Cubic Hermite Interpolating Polynomial (PCHIP) algorithm.

## Ethics Statements

The authors declare that they have read and followed the ethical requirements for publication in Data in Brief.

## CRediT authorship contribution statement

**Agbotiname Lucky Imoize:** Conceptualization, Methodology, Software, Supervision, Project administration, Funding acquisition, Writing – review & editing. **Samuel Oluwatobi Tofade:** Writing – original draft, Software, Data curation, Validation, Formal analysis, Investigation. **Glory Uzuazobona Ughegbe:** Investigation, Methodology, Data curation. **Francis Ifeanyi Anyasi:** Investigation, Methodology, Data curation. **Joseph Isabona:** Investigation, Methodology, Data curation.

## Declaration of Competing Interest

The authors declare that they have no known competing financial interests or personal relationships that could have appeared to influence the work reported in this paper.

## Data Availability

Prediction of Missing Values of Critical 4G LTE Parameters Using Experimental Dataset Derived from a Dense Urban Environment (Original data) (Mendeley Data).
